# Association Between Soft Tissue Facial Profile and Dentoskeletal Changes in High-Angle Skeletal Class II Patients After Orthodontic Treatment: A Retrospective Cephalometric Study

**DOI:** 10.3390/bioengineering13040379

**Published:** 2026-03-26

**Authors:** Kay Shuen Chan, Tingting Wei, Zhiyi Shan

**Affiliations:** 1 Division of Paediatric Dentistry and Orthodontics, Faculty of Dentistry, The University of Hong Kong, Hong Kong 999077, China; dr.kaychan@connect.hku.hk; 2 Department of Preventive Dentistry, Shanghai Ninth People’s Hospital, Shanghai Jiao Tong University School of Medicine, Shanghai 200011, China; 3 College of Stomatology, Shanghai Jiao Tong University, Shanghai 200011, China; 4 National Center for Stomatology, National Clinical Research Center for Oral Diseases, Shanghai 200011, China

**Keywords:** hyperdivergent, skeletal class II, profile changes, cephalometry, correlation, high mandibular plane angle, facial angle, *Y*-axis, lip changes, orthodontic treatment

## Abstract

Orthodontic treatment of high-angle skeletal Class II patients often leads to unsatisfactory facial profiles, highlighting the need to understand the relationship between dentoskeletal changes and soft tissue profile alterations. This retrospective study examined female Southern Chinese patients (*n* = 21, mean age 24.7 ± 4.8) with high-angle skeletal class II. Pre- and post-treatment lateral cephalograms were analysed for linear, angular, and proportional changes. Correlations between facial profile changes and dentoskeletal modifications were assessed using Pearson and Spearman’s coefficients. Results showed moderate correlations between changes in the facial angle with changes to the skeletal facial angle and skeletal *Y*-axis. A nearly perfect correlation was found between horizontal movements of the skeletal gnathion and changes in the nasolabial angle (r = 0.99, *p* < 0.001). Horizontal upper lip changes correlated with horizontal movements of upper (r = 0.70, *p* < 0.01) and lower incisors (r = 0.64, *p* < 0.01). No significant correlations were found between alterations to the incisors and changes in the facial angle or *Y*-axis. In conclusion, facial angle, *Y*-axis, and proportion changes relate closely to skeletal alterations but not to incisor positional changes, while lip changes correspond with incisor movements in high-angle skeletal Class II female patients. These findings are population-specific and require validation in male and other ethnic cohorts.

## 1. Introduction

The term “hyperdivergence” was introduced by Schudy, who linked the depth of overbites with the angle of facial divergence and facial proportions [1]. As early as 1964, Schudy emphasized that classifying patients solely by the anteroposterior (AP) relationship between the maxilla and mandible was inadequate; he argued that vertical facial dimensions must also be considered for a comprehensive diagnosis [1]. Consistent evidence indicates that hyperdivergent patients experience a poorer oral health-related quality of life compared to normodivergent individuals [2] and are more likely to report psychological distress [3], as well as psychological disability and handicap [4]. Addressing hyperdivergence, particularly in Class II patients, is therefore of significant clinical importance for multiple reasons.

Although hyperdivergent patients report no subjective difference in oral function compared to normodivergent individuals, objective assessments reveal that they have smaller masticatory muscles and weaker bite forces compared to normal and hypodivergent patients [5]. The reduced muscle strength is clinically important, as it correlates positively with both the number of occlusal contacts and masticatory efficiency [6]. Buschang further demonstrated that mandibular rotation critically influences the AP position of the chin [7]. Hyperdivergent patients generally exhibit a retrusive mandible caused by backward and downward mandibular rotation, often secondary to molar extrusion [8]. Consequently, inadequate control of the vertical dimension during orthodontic treatment may exacerbate AP skeletal discrepancies. Additionally, hyperdivergent individuals also typically display increased anterior face height and a greater anterior facial proportion [7,9]. Studies assessing layperson perceptions of facial attractiveness indicated that an increased lower facial proportion is rated significantly less attractive than either normal or decreased lower facial proportions [10,11].

Hyperdivergent skeletal Class II cases are common in orthodontic practice, representing roughly 10% of patients, yet they remain particularly challenging to treat successfully [7]. To assess the difficulty in treating Class II malocclusions, Gramling developed the Probability Index in 1995, a predictive tool based on weighted cranial and dental angular measurements. He identified a skeletal Class II relationship and a high mandibular plane angle as key predictive skeletal factors that worsen the prognosis of Class II malocclusions, and he observed that patients with these features often have poorer facial aesthetic outcomes post-treatment [12].

While orthodontic treatment historically focused on correcting dental malocclusions, the field has shifted toward a soft tissue paradigm [13], recognizing that orthodontic treatment not only improves the occlusion and the underlying bone structure, but also induces changes in the facial appearance and the smile. Among these, alterations in the smile and soft tissue profile are most perceptible to patients and laypersons, making them a crucial goal of treatment alongside dentoskeletal correction. Therefore, it is clinically relevant to understand the relationship between facial soft tissue changes and underlying dentoskeletal hard tissue changes, so that orthodontic treatment objectives can be directed toward dental and skeletal modifications that reliably produce the desired facial outcomes.

Though many studies have analysed skeletal measurements on lateral cephalograms before and after treatment across various approaches [14,15], relatively few have evaluated soft tissue changes specifically in skeletal Class II patients with high mandibular plane angles. Most research investigating the relationship between soft tissue and hard tissue changes in hyperdivergent skeletal Class II patients has been conducted on orthognathic surgery cohorts [16,17,18]. These studies typically report a near 1:1 ratio between skeletal displacement and overlying soft tissue movement, particularly in the chin region [19,20]. However, surgical patients experience immediate, large-magnitude skeletal changes, whereas orthodontic patients undergo gradual dentoskeletal adjustments over months to years, allowing greater time for soft tissue remodeling and muscular adaptation [21,22]. Consequently, soft tissue response patterns and proportionality ratios derived from surgical studies may not directly apply to patients treated with orthodontics alone [23], highlighting the need for investigations specifically examining non-surgical orthodontic cohorts such as the present study. However, because the changes after surgery are more immediate compared to those resulting from orthodontic treatment, there is likely greater opportunity for soft tissue and muscular adaptation in orthodontic patients, rendering the surgical findings less applicable to cases treated with orthodontics alone. In the limited studies on non-surgical orthodontic patients that have evaluated post-treatment soft tissue changes, the relationship between soft tissue and hard tissue changes was not examined.

Therefore, this study aims to assess the relationship between the hard tissue (dentoskeletal) changes and the soft tissue (facial profile) changes in hyperdivergent skeletal Class II patients. To minimize the influence of gender and growth-related factors, this study exclusively included female subjects with complete craniofacial growth.

## 2. Materials and Methods

### 2.1. Study Design and Ethical Approval

This is a longitudinal retrospective study with approval obtained from the Institutional Review Board of The University of Hong Kong/Hospital Authority Hong Kong West Cluster (HKU/HA HKW IRB: UW 23-633). Pre-treatment and post-treatment lateral cephalographs of female Southern Chinese patients who had completed fixed orthodontic treatment between 2010 and 2024 were retrieved from the record database of the postgraduate orthodontic clinic, Faculty of Dentistry. All radiographs were obtained using the same digital cephalometric system (Morita Co., Kyoto, Japan) under standardized exposure parameters and a consistent source-to-subject distance. Written informed consent was obtained from all patients prior to treatment, authorizing the use of their clinical records for research purposes.

### 2.2. Sample Size Calculation

Sample size calculation was conducted using G*Power software (version 3.1.9.6). An effect size of 0.707 was derived from a coefficient of determination (r^2^ = 0.5). For a two-tailed test with 95% power, a sample size of 20 participants was calculated as sufficient to detect a significant correlation. The level of statistical significance (alpha) was set at *p* < 0.05.

### 2.3. Eligibility Criteria for Subjects

The patients were screened according to the following PICO framework.

- *Population (P)*: Female Southern Chinese patients with completed craniofacial growth (CVM stage CS5 or above), high mandibular plane angle (SN-MnP > 35°), and skeletal Class II relationship (ANB > 4°).
- *Intervention (I)*: Comprehensive fixed orthodontic treatment with pre-adjusted edgewise appliances, delivered by postgraduate residents under faculty supervision. Treatment varied according to clinical presentation and included extraction/nonextraction protocols, temporary anchorage devices, and interarch elastics as indicated.
- *Comparison (C)*: Pre-treatment (T0) versus post-treatment (T1) cephalometric measurements within the same subjects.
- *Outcome (O)*: Changes in soft tissue facial profile parameters (facial angle’, *Y*-axis’, nasolabial angle, lip position, soft tissue facial proportion) and their correlation with underlying dentoskeletal changes.

Exclusion criteria comprised the presence of craniofacial syndromes, evidence of mentalis muscle strain or lip-trapping on lateral cephalograms, and poor-quality radiographic images at either pre-treatment (T0) or post-treatment (T1) time points. Poor-quality lateral cephalograms are defined as cephalograms in which the key landmarks used in this study were not clearly visible or defined. Subjects with visible mentalis muscle strain and lip-trapping in the lateral cephalograms were excluded as these features may significantly affect the position of the soft tissue pogonion, the upper lip and the labiomental fold.

### 2.4. Cephalometric Analysis and Reliability Assessment

All pre-treatment (T0) and post-treatment (T1) lateral cephalograms were imported into CASSOS (Computer Assisted Simulation System for Orthognathic Surgery) cephalometric analysis software (version 2000; SoftEnable Technology Ltd., Hong Kong, China) for digital tracing and measurement. CASSOS is an integrated PC Windows-based software package, originally developed in Hong Kong, that provides comprehensive cephalometric analysis, surgical planning, and soft tissue prediction capabilities. Prior to analysis, each image was calibrated using the known linear distance of the radiographic ruler imprinted on each cephalogram. This calibration step ensures accurate linear measurements by converting pixel coordinates to absolute millimeters based on the ruler’s known dimensions. Landmark identification and tracing were performed by a single calibrated operator (K.C.), an orthodontist with over 3 years of clinical and research experiences.

**Figure 1:**
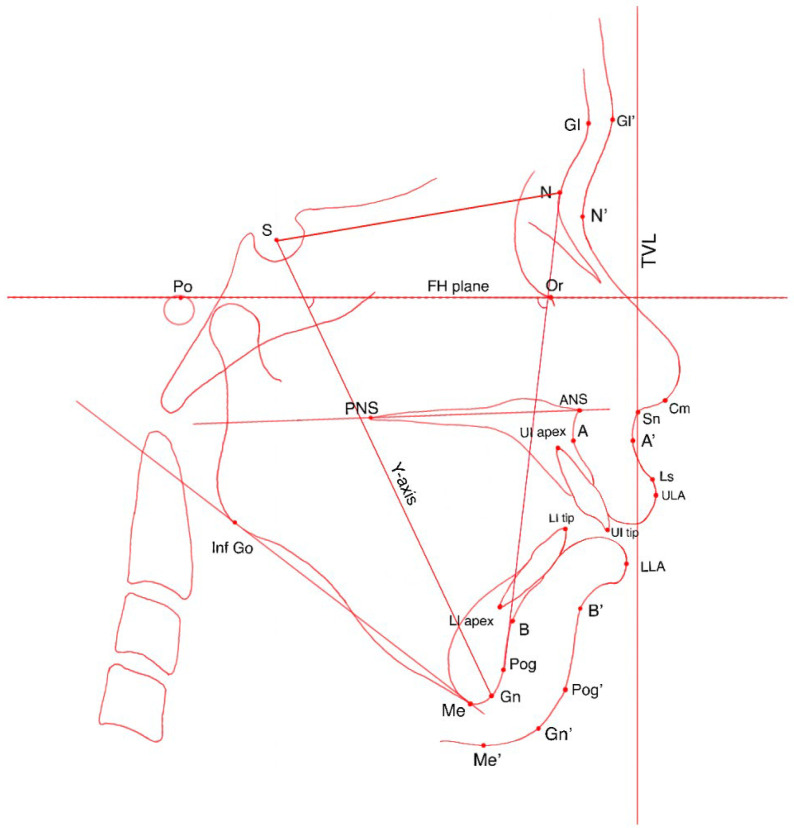
Diagram showing the landmarks used. Porion (Po), orbitale (Or), sella (S), skeletal nasion (N), soft tissue nasion (N’), Frankfort Horizontal plane (FH plane), skeletal glabella (Gl), soft tissue glabella (Gl’), posterior nasal spine (PNS), anterior nasal spine (ANS), columella (Cm), subnasale (Sn), subspinale (A), soft tissue A point (A’), labialis superiorus (Ls), upper lip most anterior point (ULA), lower lip most anterior point (LLA), skeletal B point (B), soft tissue B point (B’), skeletal pogonion (Pog), soft tissue pogonion (Pog’), skeletal gnathion (Gn), soft tissue gnathion (Gn), skeletal menton (Me), soft tissue menton (Me’), inferior gonion (Inf Go), upper incisor crown tip (UI tip), upper incisor root apex (UI apex), lower incisor crown tip (LI tip), lower incisor root apex (LI apex).

Linear measurements for each landmark were defined as its horizontal distance (in millimeters) to the True Vertical Line (TVL). The TVL is a vertical line perpendicular to the Frankfort Horizontal plane (constructed from porion to orbitale), intersecting the subnasale [24]. Specific landmarks used for tracing are detailed in Table 1 and illustrated in Figure 1. Angular, proportional, and linear measurements derived from these landmarks are presented in Table 2. For each patient, treatment changes were calculated as the difference between T1 and T0. These differences were used to examine correlations between soft tissue profile changes and underlying dentoskeletal changes.

To assess intra-operator reliability, all cephalometric tracings were repeated after a minimum washout period of one month. The original and repeated measurements were compared using two complementary approaches. First, the intraclass correlation coefficient (ICC) was calculated using a two-way mixed-effects model for absolute agreement. Second, method error was calculated using Dahlberg’s formula, ME = √(Σd^2^/2n), where d is the difference between repeated measurements and *n* is the number of duplicate measurements.

Statistical analyses were performed using SPSS (version 29.0.1.0). Paired *t*-tests were employed to evaluate within-group differences between pre-treatment (T0) and post-treatment (T1) measurements. The Shapiro–Wilk test was used to assess the normality of data distribution. To examine correlations between soft tissue profile changes and underlying dentoskeletal changes, the Pearson correlation coefficient was applied for parametrically distributed data. Where the assumption of normality was violated, the non-parametric Spearman’s rank correlation coefficient was used instead. The threshold for statistical significance was set at *p* < 0.05 (two-tailed).

## 3. Results

### 3.1. Patient Demographics and Treatment Characteristics

A total of 21 female patients between the ages of 16 and 35 years were included, with a mean age of 24.7 ± 4.80 years. All patients underwent comprehensive fixed orthodontic treatment using pre-adjusted edgewise appliances (0.022-inch slot) with MBT™ prescription bracket system (Victory Series, 3M Unitek, St. Paul, MN, USA). Treatment was delivered by postgraduate orthodontic residents under faculty supervision, with mechanotherapy individualized to each patient’s presenting malocclusion. No patients were treated with clear aligners during this period. Treatment approaches varied according to clinical presentation and included extraction protocols (four first premolars, *n* = 9; two maxillary premolars, *n* = 4; nonextraction, *n* = 8) and temporary anchorage device (TAD) utilization (*n* = 7). Mechanotherapy aimed to achieve Class I canine and molar relationships, normal overjet and overbite, and improved facial profile through a combination of space closure, torque control, and vertical dimension management. Following active treatment, all patients received fixed lingual retainers (mandibular canine-to-canine, maxillary canine-to-canine when indicated) and removable vacuum-formed retainers for full-time wear (first 12 months) followed by nighttime wear. Retention protocols were standardized across operators. Post-treatment cephalograms (T1) were obtained at debonding prior to retainer delivery. All patients exhibited an ANB angle exceeding 5 degrees and a mandibular plane angle greater than 38.5 degrees. Treatment duration ranged from 1.5 to 4.0 years, with a mean duration of 2.7 ± 0.67 years. Pre-treatment demographic and cephalometric characteristics of the sample are summarised in Table 3.

### 3.2. Changes in the Dentofacial Parameters Following Orthodontic Treatment

Intra-operator reliability for all cephalometric measurements was excellent to good. As shown in Table 4, intraclass correlation coefficients (ICC) exceeded 0.9 for all repeated measurements and demonstrated excellent intra-operator reliability, with the exception of two measurements that achieved good reliability: the horizontal distance from hard tissue pogonion (Pog) to the TVL (ICC = 0.876) and the horizontal distance from soft tissue B point (B’) to the TVL (ICC = 0.829). Normality of the data was assessed using Shapiro–Wilk tests. Most variables were normally distributed; however, deviations from normality were observed for the horizontal distance from Pog to the TVL and from B’ to the TVL.

Descriptive statistics for changes in cephalometric parameters from T0 to T1 are presented in Table 5. Skeletal changes were minimal; only a notable increase in Gn to Sn (2.18 mm ± 4.05) was observed. Dental changes were pronounced in upper and lower incisor inclination, showing substantial decreases of 7.89° ± 11.07 for UI-MxP and 4.13° ± 6.86 for LI-MnP. Soft tissue changes included a considerable increase in the nasolabial angle of 4.39° ± 8.09.

### 3.3. Correlation Between Skeletal Changes and Soft Tissue Changes

**Figure 2:**
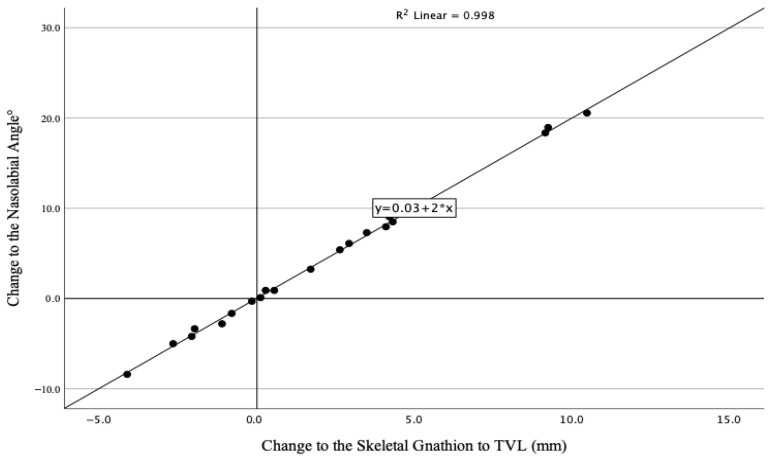
Scatter plot showing the relationship between the horizontal changes to the skeletal gnathion and changes in the nasolabial angle. The tight linear relationship (r = 0.99, *p* < 0.001) is evident, with no obvious outliers. Diagnostic analysis confirmed that no single data point exerted disproportionate influence on the correlation (maximum Cook’s distance = 0.32).

Given the deviations from normality observed for the horizontal distances from Pog to the TVL and from B’ to the TVL, Spearman’s rank correlation coefficient was used to assess relationships involving these parameters, whereas Pearson’s correlation coefficient was applied to all remaining variables. The study revealed a nearly perfect positive correlation between horizontal movements of the skeletal gnathion and changes in the nasolabial angle (r = 0.99, *p* < 0.001) (Figure 2). Horizontal shifts in the skeletal gnathion also showed strong negative correlations with horizontal changes in the upper lip (r = −0.90, *p* < 0.001), lower lip (r = −0.70, *p* < 0.001), and soft tissue A point (r = −0.79, *p* < 0.001). In contrast, changes in the skeletal pogonion and menton did not significantly correlate with angular changes in the nasolabial angle or horizontal changes in the lips, soft tissue A point, or soft tissue B point (Table 6).

To evaluate the robustness of this exceptionally strong correlation, we performed additional diagnostic analyses. Examination of standardized residuals confirmed normality (Shapiro–Wilk *p* = 0.342). Calculation of Cook’s distance identified no influential cases exceeding the conventional threshold of 1.0 (range: 0.002 to 0.32), indicating that no single observation disproportionately influenced the regression line. Visual inspection of the scatter plot (Figure 2) confirmed a tight linear relationship without obvious outliers. These analyses suggest that while the correlation is remarkably strong, it is not an artifact of data errors or undue influence from extreme values.

Moderate correlations were found between the skeletal and soft tissue *Y*-axes (r = 0.75, *p* < 0.001), skeletal *Y*-axis and soft tissue facial angle (r = −0.77, *p* < 0.001), skeletal facial angle and soft tissue *Y*-axis (r = −0.66, *p* < 0.001), and skeletal facial angle and soft tissue facial angle (r = −0.74, *p* < 0.001). Changes in the hard tissue facial proportion moderately correlated with changes in the soft tissue facial proportion (r = 0.56, *p* < 0.05). Additionally, soft tissue facial angle changes correlated with horizontal movements of the hard tissue pogonion (r = 0.55, *p* < 0.05) and menton (r = 0.45, *p* < 0.05), but not with the hard tissue gnathion (r = 0.07, *p* = 0.778).

### 3.4. Correlation Between Dental Changes and Soft Tissue Changes

A significant association was observed between dental changes and the corresponding soft tissue modifications following fixed appliance therapy. Specifically, dental positional and angular changes demonstrated notable correlations with changes in lip position and certain soft tissue landmarks. Changes to the horizontal position of the upper incisors were significantly and positively correlated with horizontal changes in the upper lip (r = 0.70, *p* < 0.001), lower lip (r = 0.60, *p* < 0.05), and the soft tissue A point (r = 0.77, *p* < 0.001). Modifications to the horizontal position of the lower incisors exhibited significant correlations with horizontal changes in the upper lip (r = 0.68, *p* < 0.05) and lower lip (r = 0.56, *p* < 0.05). However, no correlation was identified between changes to the horizontal position of the lower incisors, and horizontal changes in the soft tissue B point.

Angular changes to the upper incisors were significantly correlated with the horizontal position of the upper lip (r = 0.64, *p* < 0.05), lower lip (r = 0.62, *p* < 0.05), soft tissue A point (r = 0.57, *p* < 0.05) and angular changes in the nasolabial angle (r = −0.53, *p* < 0.05). Angular changes in the upper incisors significantly correlated with horizontal changes in the upper lip (r = 0.64, *p* < 0.05), lower lip (r = 0.62, *p* < 0.05), soft tissue A point (r = 0.57, *p* < 0.05), and angular changes in the nasolabial angle (r = −0.53, *p* < 0.05). Angular changes in the lower incisors correlated significantly with horizontal changes in the upper lip (r = 0.50, *p* < 0.05) but showed only a weak, non-significant correlation with the lower lip (r = 0.40, *p* = 0.407).

Except for a significant correlation between lower incisor angular changes and the soft tissue facial axis (r = −0.51, *p* < 0.05), no other dental changes showed significant correlations with the soft tissue facial axis or facial angle. Additional scatter plots of the data are presented in Appendix A.

## 4. Discussion

This study investigated the relationship between dentoskeletal changes and soft tissue facial profile modifications in a clinically challenging population: adult female Southern Chinese patients with high-angle skeletal Class II malocclusion treated with orthodontics alone. These patients are particularly difficult to manage and face elevated risk of unsatisfactory aesthetic outcomes, making the identification of predictable soft tissue responses especially valuable. Our findings demonstrate significant correlations between skeletal angular changes and soft tissue profile alterations, most notably in the facial angle and *Y*-axis measurements. These results corroborate previous research but extend its applicability by confirming that, even in non-surgical orthodontic cohorts—where skeletal changes are more gradual and modest in magnitude—modifications to underlying bony structures are reliably reflected in the overlying facial soft tissues. However, the varying strength of these correlations underscores that soft tissue response is not uniform and may be modulated by individual factors such as tissue thickness, tonicity, and adaptive capacity. From a clinical standpoint, these findings provide evidence-based guidance for treatment planning: interventions that improve skeletal relationships can be expected to yield corresponding enhancements in facial profile, though the degree of response will vary among patients.

This study determined that the ratio of upper lip retraction to upper incisor retraction in adult hyperdivergent skeletal Class II patients is approximately 0.4. This value is consistent with findings from a study investigating Vietnamese adults with convex facial profiles, which reported a ratio of 0.435 at the incisal edge [25]. Moreover, the approximate 0.4 ratio closely aligns with reports in younger Class II division 1 patients aged 8 to 16 years old [26]. These consistent ratios across different age groups suggest a stable relationship between upper incisor movement and corresponding upper lip response in Class II malocclusion cases. This study also found that changes in the lower lip showed a higher correlation with changes to the upper incisors than with changes to the lower incisors. This reflects the observation that the lower lip rests on the incisal third to the upper incisors and the lower lip sagittal position relates more closely with the upper incisors sagittal position than the lower incisors. The nasolabial angle to upper incisor retraction ratio reported in Class II division 1 adolescents was −1.63 [26]. In contrast, our study observed a substantially larger ratio of −7.29 in adult patients. This marked difference may be attributed to age-related variations in soft tissue response. Supporting this, recent studies comparing hyperdivergent Class II division 1 patients found that changes in the nasolabial angle could be more pronounced in adults than in adolescents [27,28]. This phenomenon could be partially explained by alterations in upper lip thickness during treatment; while lip thickness tends to increase throughout adolescence, it generally decreases during adulthood [29,30]. Consequently, the reduction in lip thickness in adults may amplify the nasolabial angle changes associated with upper incisor retraction, as thinner, soft tissues offer less resistance to positional changes and transmit a greater proportion of dental movement to the overlying lip and nasal base structures.

In the present study, no significant correlation was observed between alterations in skeletal facial proportions and sagittal positional changes in the soft tissue B point and soft tissue Pogonion. Contrary to theoretical expectations that the B point would exhibit anterior displacement in response to counterclockwise mandibular rotation, the data did not support this hypothesis. This finding aligns with a case report [31] that showed no anterior movement of chin after counterclockwise rotation of the mandible and the CBCT superimposition showed a posterior displacement of the mandible. Furthermore, a more recent study published in 2025 [32] identified that female patients and those classified as skeletal Class II exhibited significantly increased likelihoods of unexpected condylar displacement following orthodontic intervention. These findings collectively suggest that sagittal soft tissue landmarks may not reliably reflect underlying skeletal changes, highlighting the complexity of mandibular positional adaptations post-treatment.

In orthognathic patients, movements of the soft tissue pogonion and soft tissue B point closely mirror their corresponding skeletal landmarks, typically exhibiting an almost 1:1 ratio [19,20,33]. In contrast, our study found that changes in soft tissue chin landmarks did not demonstrate significant correlations with movements of their skeletal counterparts—namely, the skeletal menton, gnathion, and pogonion—except for a moderate correlation between horizontal displacement of the soft tissue gnathion and sagittal changes in the skeletal menton (r = 0.56, *p* = 0.01). Several factors may account for this discrepancy. Firstly, orthodontic patients generally experience more gradual skeletal adjustments, allowing greater time for soft tissue remodeling and adaptation [23]. Secondly, the magnitude of skeletal discrepancy in orthognathic surgery patients is often substantially greater than that observed in orthodontic cases, leading to more pronounced and directly corresponding soft tissue changes post-surgery [17]. Lastly, the relatively small sample size in this study may limit the statistical power necessary to detect subtle soft tissue changes in the chin region. Future studies with larger cohorts are warranted to further elucidate these relationships.

The moderate to strong correlations observed between changes in the skeletal facial angle and soft tissue facial angle (r = −0.74, *p* < 0.001), as well as between skeletal and soft tissue *Y*-axis (r = 0.75, *p* < 0.001), suggest that vertical and sagittal skeletal adjustments are reflected in the overlying soft tissues. This is consistent with the biomechanical understanding that soft tissue drapes over the skeletal framework, adapting to underlying bony repositioning over time. However, the absence of significant correlations between incisor positional/angular changes and facial angle or *Y*-axis changes indicates that dental movements alone may not substantially influence overall facial profile metrics in high-angle skeletal Class II patients. Conversely, significant correlations between horizontal changes in upper and lower incisors and corresponding lip position changes (r = 0.70 and 0.64, respectively, *p* < 0.01) highlight the direct impact of dental movements on perioral soft tissues. This finding aligns with clinical observations that lip posture and contour are sensitive to anterior tooth positioning, which is critical for aesthetic outcomes in orthodontics [25,34]. The fair correlation between hard and soft tissue facial proportions (r = 0.56, *p* < 0.05) further emphasizes that while skeletal changes contribute to facial proportions, soft tissue response may be modulated by individual variability in soft tissue thickness, elasticity, and muscular adaptation [35,36]. These factors may account for the less than perfect correlation coefficients observed.

Our findings show that sagittal changes in the skeletal gnathion have a nearly perfect positive correlation with changes in the nasolabial angle (r = 0.99, *p* < 0.001) (Figure 2) and a very strong negative correlation with sagittal changes in upper lip position (r = −0.90, *p* < 0.001). While this exceptionally high correlation warrants scrutiny, diagnostic analyses confirmed no influential outliers and residuals were normally distributed, indicating the association is not an artifact of data errors or extreme values. Nevertheless, this finding should be interpreted cautiously, as the correlation magnitude exceeds what is typical in biological systems and may be amplified by the modest sample size (*n* = 21). More importantly, direct biomechanical coupling between gnathion position and nasolabial angle is anatomically implausible given the intervening soft tissues separating these landmarks. Several mechanistic explanations may account for these observed correlations. First, the “face-driven treatment” hypothesis proposes that these correlations reflect intentional treatment planning tailored to pretreatment soft tissue characteristics [8,13]. In patients presenting with an acute nasolabial angle, upper lip retraction may have been prioritized through extraction protocols with skeletal anchorage, simultaneously permitting forward mandibular adaptation and explaining the association between gnathion advancement and increased nasolabial angle [8]. Conversely, in patients with obtuse pretreatment nasolabial angles, non-extraction approaches with inter-arch elastics may have been preferred, potentially yielding gnathion advancement without concomitant lip retraction. Second, biomechanical factors may contribute directly to this relationship. Anterior displacement of the skeletal gnathion could physically stretch the overlying soft tissue envelope, with tension transmitted superiorly through the labiomental fold to the lower lip. This mechanism might subtly influence lip position, particularly in patients with thinner soft tissues. Third, concomitant vertical changes accompanying mandibular rotation—common in high-angle patients—may independently affect lip posture and competence, influencing measurements of the most anterior lip point relative to the True Vertical Line. Fourth, incisor position may serve as a mediating variable, as evidenced by significant correlations between upper incisor retraction and both forward gnathion movement (r = −0.57, *p* < 0.01) and lip retraction (r = 0.70, *p* < 0.001). Treatment mechanics that simultaneously advance the mandible and retract incisors to correct overjet could produce correlated gnathion and lip changes, with incisor movement as the primary driver of lip position.

**Figure A1:**
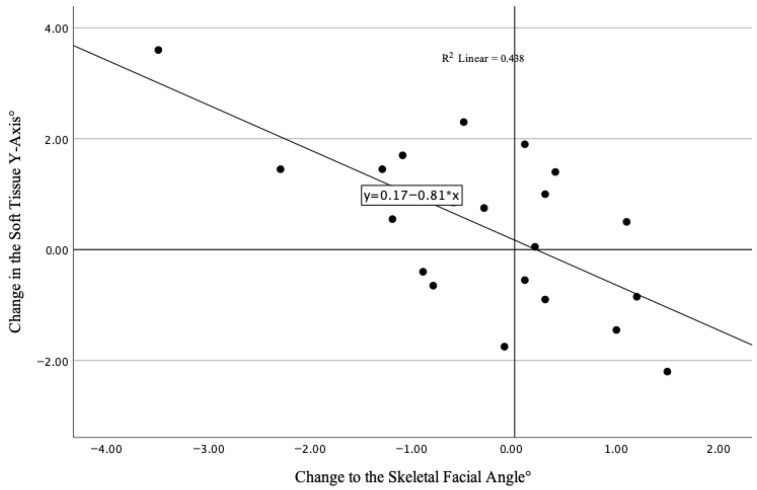
Scatter plot showing the relationship between the changes to the skeletal facial angle and changes in the soft tissue *Y*-axis.

**Figure A2:**
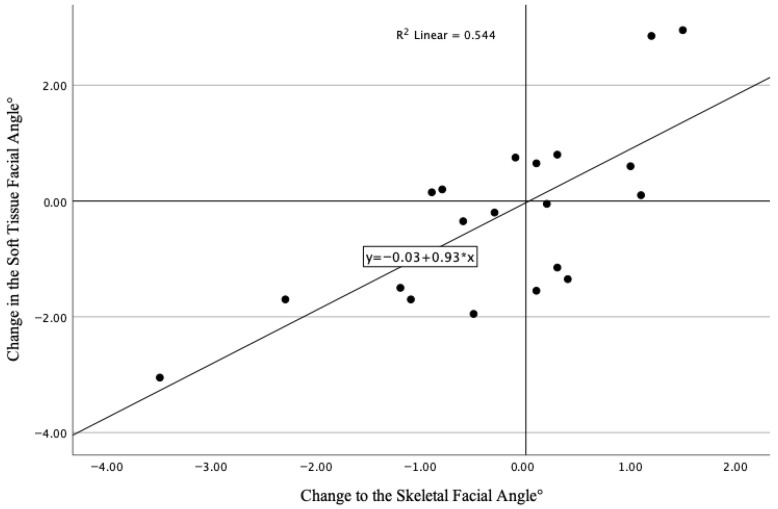
Scatter plot showing the relationship between the changes to the skeletal facial angle and changes in the soft tissue facial angle.

**Figure A3:**
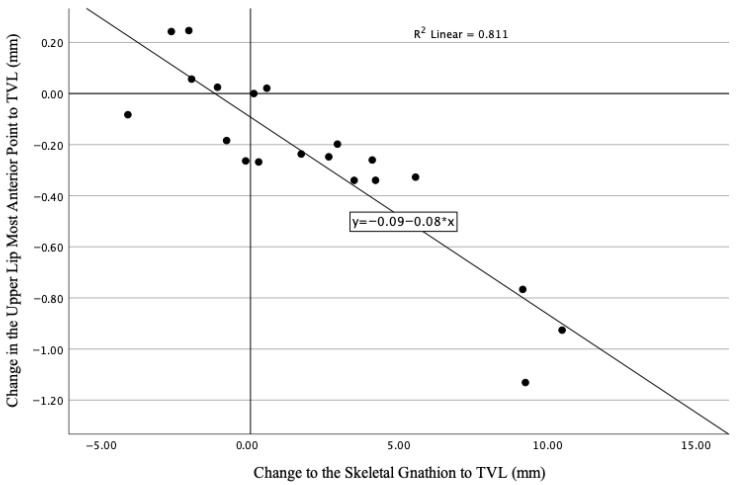
Scatter plot showing the relationship between the horizontal changes to the skeletal gnathion and horizontal changes in the most anterior point of the upper lip (ULA).

**Figure A4:**
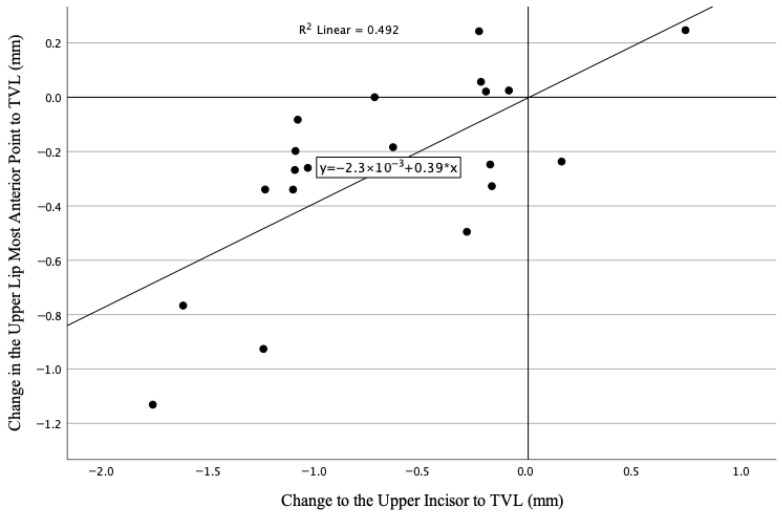
Scatter plot showing the relationship between the horizontal changes to the upper incisor crown tip and horizontal changes in the most anterior point of the upper lip (ULA).

**Figure A5:**
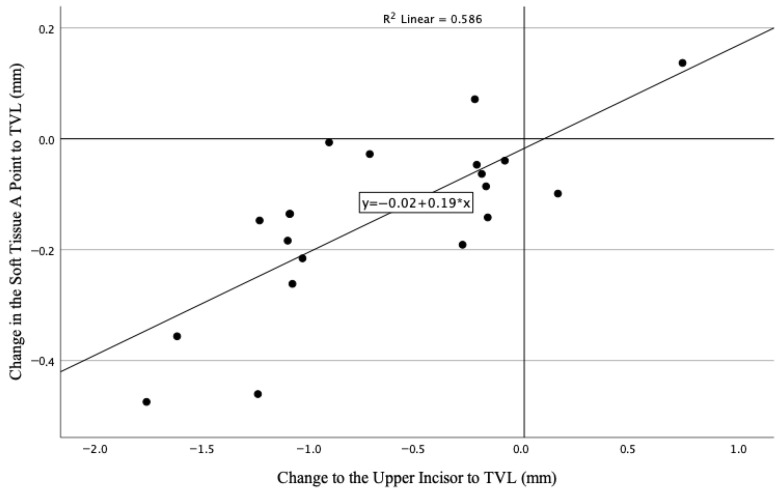
Scatter plot showing the relationship between the horizontal changes to the upper incisor crown tip and horizontal changes in the soft tissue A point (Subspinale).

**Figure A6:**
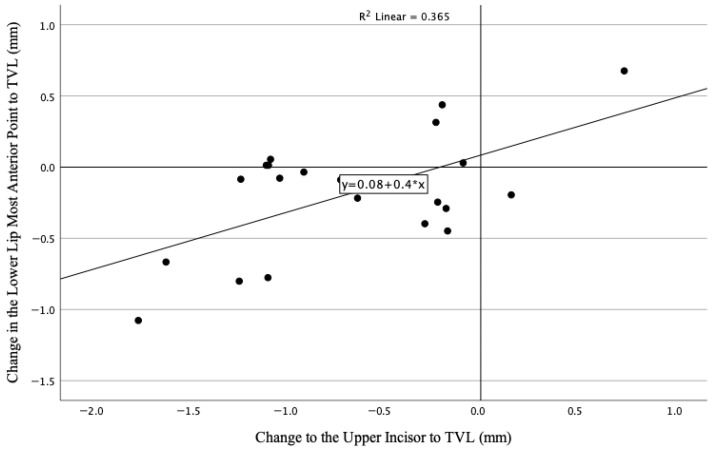
Scatter plot showing the relationship between the horizontal changes to the upper incisor crown tip and horizontal changes in the most anterior point of the lower lip (LLA).

Examination of the supplementary scatter plots (Appendix A Figure A1, Figure A2, Figure A3, Figure A4, Figure A5 and Figure A6) reveals that despite statistically significant correlations, considerable dispersion of data points is evident for several associations. For instance, while the correlation between upper incisor retraction and upper lip retraction reached statistical significance (r = 0.70, *p* < 0.001) (Figure A4), individual responses varied substantially, with some patients exhibiting minimal lip change despite substantial incisor movement. This dispersion reflects the multifactorial nature of soft tissue response, which is influenced by individual variations in soft tissue thickness [35], tonicity, age-related changes in tissue elasticity [36,37], and pretreatment lip posture [34]. The variability underscores that population-level correlations, while informative for understanding general trends, have limited utility for precise individual-level prediction. Clinicians should therefore exercise caution when applying these average ratios to individual patients, as the range of possible soft tissue responses remains wide even when correlations are statistically significant.

This study has several limitations that should be considered when interpreting the findings. First, the exclusive inclusion of female patients, while intentional to minimize gender-related confounding, limits generalizability to male patients. This decision was informed by evidence of inherent sex differences in facial soft tissue thickness and orthodontic treatment response [38]. Males exhibit greater soft tissue thickness in the lips and chin [39], which may camouflage underlying dental or skeletal changes and attenuate post-treatment profile alterations. Consequently, male patients with high-angle skeletal Class II malocclusion may require more substantial skeletal corrections to achieve comparable soft tissue improvements, and future studies specifically examining this population are warranted. Second, the modest sample size (*n* = 21), while adequately powered for the primary correlation analyses, may limit the stability of correlation estimates and precludes subgroup analyses based on specific treatment mechanics. Third, the retrospective design and lack of treatment standardization mean that observed correlations may reflect individualized clinical decision-making rather than direct biomechanical relationships, as discussed above. Fourth, the use of two-dimensional cephalometry, despite high intra-operator reliability, cannot capture three-dimensional soft tissue changes or asymmetries that may influence facial profile outcomes. Given these limitations, future studies addressing these limitations should include larger, more diverse populations encompassing both sexes, employ prospective designs with standardized treatment protocols, and utilize three-dimensional imaging to provide more comprehensive insights into the complex interplay between skeletal, dental, and soft tissue changes in hyperdivergent Class II patients.

In summary, this study demonstrates that in female Southern Chinese patients with high-angle skeletal Class II malocclusion treated orthodontically, changes in facial angle, *Y*-axis, and facial proportion correlate significantly with corresponding skeletal angular changes but not with incisor positional or angular changes. Horizontal and angular movements of the incisors are significantly associated with changes in lip position, underscoring the influence of dental movements on perioral soft tissues. These findings highlight the importance of considering both skeletal and dental factors when predicting soft tissue profile outcomes in treatment planning.

## 5. Conclusions

In female Southern Chinese patients with high-angle skeletal Class II malocclusion treated orthodontically, changes in facial angle, facial *Y*-axis, and facial proportion exhibit significant correlations with corresponding skeletal angular changes but not with incisor positional or angular changes. Horizontal and angular movements of the upper and lower incisors are significantly associated with changes in lip position, underscoring the influence of dental movements on perioral soft tissues. These findings highlight the importance of considering both skeletal and dental factors when predicting soft tissue profile outcomes in orthodontic treatment planning for this patient population.

## Figures and Tables

**Table 1:** Description of Lateral Cephalometric Landmarks.

Abbreviation	Landmark	Description
**Skeletal**		
S	Sella	The midpoint of the sella turcica
N	Nasion	The most anterior limit of the fronto-nasal suture
Po	Anatomical porion	The most superior point of the external auditory meatus
Or	Orbitale	The most inferior point on the infraorbital rim
ANS	Anterior nasal spine	The most anterior tip of the median sharp bony process of the anterior nasal spine
PNS	Posterior nasal spine	The most posterior tip of the posterior nasal spine
A	Subspinale	The most posterior point on the curvature of the concave anterior border of the maxillary alveolar process
B	Supramentale	The most posterior point on the curvature of the anterior contour of the mandible
Pog	Pogonion	The most anterior point on the mandibular symphysis
Gn	Gnathion	The most antero-inferior point of the mandibular symphysis
Me	Menton	The most inferior point on the mandibular symphysis
Inf Go	Inferior gonion	Most inferior point on the curvature of the angle of ramus
**Dental**		
UI tip	Upper incisor crown tip	Tip of the upper incisor crown
UI apex	Upper incisor root tip	Apex of the upper incisor root
LI tip	Lower incisor crown tip	Tip of the lower incisor crown
LI apex	Lower incisor root tip	Apex of the lower incisor root
**Soft Tissue**		
Gl’	Soft tissue glabella	The most anterior point of the soft tissue forehead when in natural head position.
N’	Soft tissue nasion	The most posterior point on the curvature of the nose between the forehead and the nose bridge.
Sn	Subnasale	The intersection between the inferior border of the nose and the upper lip
TVL	True vertical line	Vertical line perpendicular to the Frankfort Horizontal line, intersecting the subnasale
Cm	Columella	The most anterior-inferior point on the lower surface of the nose, determining the anterior limit of the nasolabial angle
A’	Soft tissue A point	The deepest point of the curvature between the subnasale and the lip
B’	Soft tissue B point	The deepest point on the curvature between the lower lip and the soft tissue pogonion.
Ls	Labialis superioris	The most antero-superior point of the vermilion border of the upper lip
ULA	Upper lip anterior	Upper lip most anterior point
LLA	Lower lip anterior	Lower lip most anterior point
Pog’	Soft tissue pogonion	The most anterior point on the soft tissue chin
Me’	Soft tissue menton	The most inferior point of soft tissue contour of the chin
Gn’	Soft tissue gnathion	The most antero-inferior point of the soft tissue contour of the chin
TVL	True vertical line	Line perpendicular to the Frankfort Horizontal, intersecting the Subnasale, Sn

**Table 2:** Measurements Derived from Landmarks.

Variable	Definition
**Skeletal**	
ANB (°)	Angle formed between A-N and N-B
SN-MnP (°)	Angle formed between S-N and the mandibular plane (Inferior Gonion-Menton)
*Y*-Axis (°)	Angle formed between the Frankfurt Horizontal (Porion-Orbitale) and S-Gn
Facial Angle (°)	Angle formed between the Frankfurt Horizontal (Porion-Orbitale) and N-Pog
HTFP (%)	Hard tissue lower anterior facial proportion. Vertical distance between ANS-Me/Vertical distance between Gl-Me
A to TVL (mm)	Horizontal distance between point A and the TVL
B to TVL (mm)	Horizontal distance between point B and the TVL
Pog to TVL (mm)	Horizontal distance between the pogonion and the TVL
Gn to TVL (mm)	Horizontal distance between the gnathion and the TVL
Me to TVL (mm)	Horizontal distance between the menton and the TVL
**Dental**	
UI-MxP (°)	Angle formed between the upper incisor (UI tip-UI apex) and the maxillary plane (ANS-PNS)
LI-MnP (°)	Angle formed between the lower incisor (LI tip-LI apex) and the mandibular plane (Inferior Gonion-Menton)
UI to TVL (mm)	Horizontal distance between upper incisor crown tip and the TVL
LI to TVL (mm)	Horizontal distance between lower incisor crown tip and the TVL
**Soft Tissue**	
*Y*-axis’ (°)	Angle formed between the Frankfurt Horizontal (Porion-Orbitale) and S-Gn’
Facial Angle’ (°)	Angle formed between the Frankfurt Horizontal (Porion-Orbitale) and N’-Pog’
STFP (%)	Soft tissue lower anterior facial proportion. Vertical distance between Sn-Me’/Vertical distance between Gl’-Me’
A’ to TVL (mm)	Horizontal distance between point A’ and the TVL
B’ to TVL (mm)	Horizontal distance between the point B’ and the TVL
Pog’ to TVL (mm)	Horizontal distance between the soft tissue pogonion, Pog’ and the TVL
Gn’ to TVL (mm)	Horizontal distance between the soft tissue gnathion, Gn’ and the TVL
Me’ to TVL (mm)	Horizontal distance between the soft tissue menton, Me’ and the TVL
ULA to TVL (mm)	Horizontal distance between ULA and the TVL
LLA to TVL (mm)	Horizontal distance between LLA and the TVL
NLA (°)	Nasolabial angle. Angle formed between Cm-Sn and Sn-Ls

**Table 3:** Descriptive statistics of subjects before treatment (T0).

Characteristics	Mean ± SD
ANB (°)	6.5 ± 1.04
Wits appraisal (mm)	−1.24 ± 0.34
SN-MP (°)	43.96 ± 3.58
FH-MnP (°)	34.76 ± 3.46
MMPA (°)	32.61 ± 4.71
Age (years)	24.7 ± 4.80

**Table 4:** Intra-operator reliability for cephalometric measurements.

Measurements	Intraclass Correlation Coefficient (ICC)	Dahlberg’s Error
ANB (°)	0.938	0.41°
SN-MnP (°)	0.991	0.32°
*Y*-Axis (°)	0.993	0.28°
Facial Angle (°)	0.991	0.31°
A to TVL (mm)	0.973	0.24 mm
B to TVL (mm)	0.971	0.26 mm
Pog to TVL (mm)	0.876	0.48 mm
Gn to TVL (mm)	0.977	0.22 mm
Me to TVL (mm)	0.978	0.21 mm
UI to TVL (mm)	0.996	0.34%
UI-MxP (°)	0.995	0.18 mm
LI to TVL (mm)	0.993	0.21°
LI-MnP (°)	0.987	0.19 mm
HTFP (%)	0.981	0.32°
*Y*-axis’ (°)	0.976	0.43°
Facial Angle’ (°)	0.984	0.39°
A’ to TVL (mm)	0.974	0.26 mm
B’ to TVL (mm)	0.829	0.54 mm
Pog’ to TVL (mm)	0.967	0.31 mm
Gn’ to TVL (mm)	0.968	0.29 mm
Me’ to TVL (mm)	0.951	0.36 mm
ULA to TVL (mm)	0.988	0.23 mm
LLA to TVL (mm)	0.992	0.21 mm
NLA (°)	0.989	0.54°
STFP (%)	0.984	0.41%

**Table 5:** Changes in the cephalometric parameters from T0 to T1.

Changes (T1-T0)	Measurements	Mean ± SD
**Skeletal **		
Angular	ANB (°)	−0.23 ± 1.40
	SN-MnP (°)	0.84 ± 1.42
	*Y*-Axis (°)	0.49 ± 1.30
	Facial Angle (°)	−0.30 ± 1.19
Linear	A to Sn (mm)	0.01 ± 0.14
	B to Sn (mm)	0.06 ± 0.30
	Pog to Sn (mm)	0.11 ± 0.46
	Gn to Sn (mm)	2.18 ± 4.05
	Me to Sn (mm)	−0.05 ± 0.30
Proportional	HTFP (%)	0.12 ± 0.71
**Dental**		
Angular	UI-MxP (°)	−7.89 ± 11.07
	LI-MnP (°)	−4.13 ± 6.86
Linear	UI to Sn (mm)	−0.67 ± 0.63
	LI to Sn (mm)	−0.36 ± 0.50
**Soft Tissue**		
Angular	*Y*-axis’ (°)	0.42 ± 1.46
	Facial Angle’ (°)	−0.31 ± 1.50
	NLA (°)	4.39 ± 8.09
Linear	A’ to Sn (mm)	−0.14 ± 0.15
	B’ to Sn (mm)	0.17 ± 0.46
	Pog’ to Sn (mm)	−0.01 ± 0.50
	Gn’ to Sn (mm)	0.01 ± 0.56
	Me’ to Sn (mm)	−0.09 ± 0.80
	ULA to Sn (mm)	−0.26 ± 0.35
	LLA to Sn (mm)	−0.18 ± 0.42
Proportional	STFP (%)	−0.03 ± 1.26

**Table 6:** Correlation between skeletal changes and soft tissue changes.

	r Values	Skeletal								Dental			
		*Y*-Axis (°)	Facial Angle (°)	HTFP (%)	A (mm)	B (mm)	Pog (mm)	Gn (mm)	Me (mm)	UI to Sn (mm)	UI-MxP (°)	LI to Sn (mm)	LI-MnP (°)
**Facial profile**	*Y*-axis’ (°)	0.75 **	−0.66 **	−0.05	−0.22	−0.09	*−0.19*	0.05	−0.4	−0.36	−0.4	−0.06	−0.27
	Facial Angle’ (°)	−0.77 **	0.74 **	0.05	0.17	0.13	*0.35*	0.07	0.45 *	0.24	0.36	0.04	0.17
	STFP (%)	−0.4	0.48 *	0.56 **	0.16	0.22	*0.42*	0.01	0.1	0.13	−0.2	0.36	0.26
	A’ (mm)	−0.32	0.26	0.24	0.33	0.23	*0.24*	−0.79 **	0.06	0.77 **	0.57 **	0.57 **	0.65 **
	B’ (mm)	*−0.35*	*0.17*	*0.26*	*0.06*	*0.34*	*0.35*	*−0.43*	*0.13*	*0.33*	0.49 *	*0.29*	*0.21*
	Pog’ (mm)	−0.21	0.26	−0.26	−0.09	0.11	*0.37*	−0.05	0.33	0.02	0.18	−0.44 *	−0.16
	Gn’ (mm)	−0.41	0.41	−0.29	0.22	0.26	*0.27*	0	0.56 **	0.24	0.38	−0.26	0.07
	Me’ (mm)	−0.31	0.3	−0.35	0.09	0.08	*0.07*	0.03	0.36	0.06	0.17	−0.17	0
**Lips**	ULA (mm)	−0.29	0.24	0.04	0.35	0.31	*0.17*	−0.90 **	0.06	0.70 **	0.64 **	0.50 *	0.68 **
	LLA (mm)	−0.38	0.31	−0.02	0.24	0.19	*0.19*	−0.70 **	0.15	0.60 **	0.62 **	0.41	0.56 **
	NLA (°)	0.03	0	0.05	−0.16	−0.28	*−0.02*	0.99 **	0.08	−0.57 **	−0.53 *	−0.33	−0.56 **

*. Correlation is significant at the 0.05 level (2-tailed). **. Correlation is significant at the 0.01 level (2-tailed). **Data in bold highlights correlations showing significance at both 0.05 and 0.01 level**. *Data in italics is non-parametric and refers to Spearman’s rank correlation.*

## Data Availability

The data presented in this study are available upon request from the corresponding author. The data are not publicly available due to privacy limitations.

## Appendix A

Supplemental information included in the Appendix include the normality tests and supplemental scatter plots to demonstrate the correlations between several of the dentoskeletal changes with soft tissue changes.

**Table A1:** Shapiro–Wilk Test for Normality.

	Shapiro–Wilk	*p*-Value
ANB (°)	0.952	0.372
SN-MnP (°)	0.903	0.041
*Y*-Axis (°)	0.935	0.171
Facial Angle (°)	0.944	0.266
HTFP (%)	0.980	0.932
A to Sn (mm)	0.946	0.286
B to Sn (mm)	0.940	0.219
Pog to Sn (mm)	0.845	0.004
Gn to Sn (mm)	0.947	0.298
Me to Sn (mm)	0.931	0.146
UI to Sn (mm)	0.953	0.390
UI-MxP (°)	0.970	0.726
LI to Sn (mm)	0.894	0.057
LI-MnP (°)	0.941	0.232
*Y*-axis’ (°)	0.982	0.955
Facial Angle’ (°)	0.943	0.249
STFP (%)	0.968	0.681
A’ to Sn (mm)	0.944	0.266
B’ to Sn (mm)	0.847	0.004
Pog’ to Sn (mm)	0.942	0.235
Gn’ to Sn (mm)	0.982	0.948
Me’ to Sn (mm)	0.971	0.754
ULA to Sn (mm)	0.912	0.060
LLA to Sn (mm)	0.971	0.755
NLA (°)	0.944	0.266
